# One-stage posterior approaches for treatment of thoracic spinal infection: Transforaminal and costotransversectomy, compared with anterior approach with posterior instrumentation

**DOI:** 10.1097/MD.0000000000008352

**Published:** 2017-10-20

**Authors:** Fu-Cheng Kao, Tsung-Ting Tsai, Chi-Chien Niu, Po-Liang Lai, Lih-Huei Chen, Wen-Jer Chen

**Affiliations:** Department of Orthopaedic Surgery, Spine Section, Bone and Joint Research Center, Chang Gung Memorial Hospital and Chang Gung University College of Medicine, Taoyuan, Taiwan.

**Keywords:** 1-stage posterior approach, costotransversectomy, thoracic spondylodiscitis, transforaminal thoracic interbody fusion

## Abstract

Treating thoracic infective spondylodiscitis with anterior surgical approaches carry a relatively high risk of perioperative and postoperative complications. Posterior approaches have been reported to result in lower complication rates than anterior procedures, but more evidence is needed to demonstrate the safety and efficacy of 1-stage posterior approaches for treating infectious thoracic spondylodiscitis.

Preoperative and postoperative clinical data, of 18 patients who underwent 2 types of 1-stage posterior procedures, costotransversectomy and transforaminal thoracic interbody debridement and fusion and 7 patients who underwent anterior debridement and reconstruction with posterior instrumentation, were retrospectively assessed.

The clinical outcomes of patients treated with 1-stage posterior approaches were generally good, with good infection control, back pain relief, kyphotic angle correction, and either partial or solid union for fusion status. Furthermore, they achieved shorter surgical time, fewer postoperative complications, and shorter hospital stay than the patients underwent anterior debridement with posterior instrumentation.

The results suggested that treating thoracic spondylodiscitis with a single-stage posterior approach might prevent postoperative complications and avoid respiratory problems associated with anterior approaches. Single-stage posterior approaches would be recommended for thoracic spine infection, especially for patients with medical comorbidities.

## 1 Introduction

The thoracic vertebrae are the most common site of nonpyogenic spondylodiscitis, and the second most common site of pyogenic spinal infections.^[1,2]^ Most cases of infectious thoracic spondylodiscitis can be managed nonsurgically with antibiotics for a minimum of 4 to 6 weeks, and immobilization with a brace or other forms of supportive care.^[3]^ Indications for surgery include neurological deficits and spinal instability with risk of injury to neurological structures, and relative indications for surgery include infection resulting from an unknown pathogen, poorly controlled infection, and intractable pain.

Surgical interventions for thoracic spondylodiscitis remain challenging for spine surgeons, especially for patients with comorbidities such as diabetes mellitus, immunosuppression, intravenous drug use, alcoholism, liver cirrhosis, malignancy, and renal failure. The goals of surgical treatment of thoracic spinal infections are to debride the infected foci, identify the pathogen, decompress the neural elements, and restabilize the deformed spine. Traditionally, anterior procedures have been shown to achieve more radical debridement and direct reconstruction with fusion. However, the standard anterior approach to the thoracic spine, that is, thoracotomy, requires the placement of a chest tube that carries a relatively high risk of postoperative pneumonia and lung atelectasis due to impaired respiratory function.^[4]^

Posterior instrumentation following anterior debridement and reconstruction, on the contrary, is staged or performed in a sequential manner during a single anesthesia session and is dependent on the stability of bony structures. Staged operations may result in spinal instability between the stages, and complications from the first stage may lead to postponement or abandonment of the second stage.^[5]^ Operations staged sequentially within a single anesthesia session increase operating time and blood loss, which may increase the risk of complications, especially in elderly patients with medical comorbidities. Whereas, 1-stage posterior procedures with instrumentation can stabilize the spine and correct kyphotic deformity, and have been shown to have a lower complication rate than anterior procedures.^[6]^

The purpose of this study was to investigate the safety and efficacy of 2 different single-stage posterior approaches for treating infectious thoracic spondylodiscitis, a costotransversectomy approach for thoracic interbody debridement and fusion with pedicle screw instrumentation and transforaminal thoracic interbody debridement and fusion (TTIDF) with pedicle screw instrumentation by comparing their treatment outcomes to anterior approach with posterior instrumentation.

## 2 Methods

Among all spine surgeries performed between November 2009 and October 2014, patients were included in this retrospective study if they received posterior pedicle screw instrumentation for pyogenic thoracic spondylodiscitis and followed-up for at least 12 months after surgery. Medical records, including imaging studies, laboratory data, neurological function data, and functional outcomes were reviewed and analyzed. Indications for surgery were pyogenic thoracic spondylodiscitis with spinal instability due to progressive bony destruction, intractable back pain, unknown pathogens, and poor response to medical treatment. Exclusion criteria included following up less than 12 months, patients without anterior or posterior debridement and reconstruction, multilevel spinal infections without severe instability, and complications from previous spine surgeries or spine trauma. The present study was approved by the Institutional Review Board (IRB) of Chang Gung Medical Foundation on November 12, 2013. The IRB is organized and operates according to Good Clinical Practice and the applicable laws and regulations.

**Figure 1:**
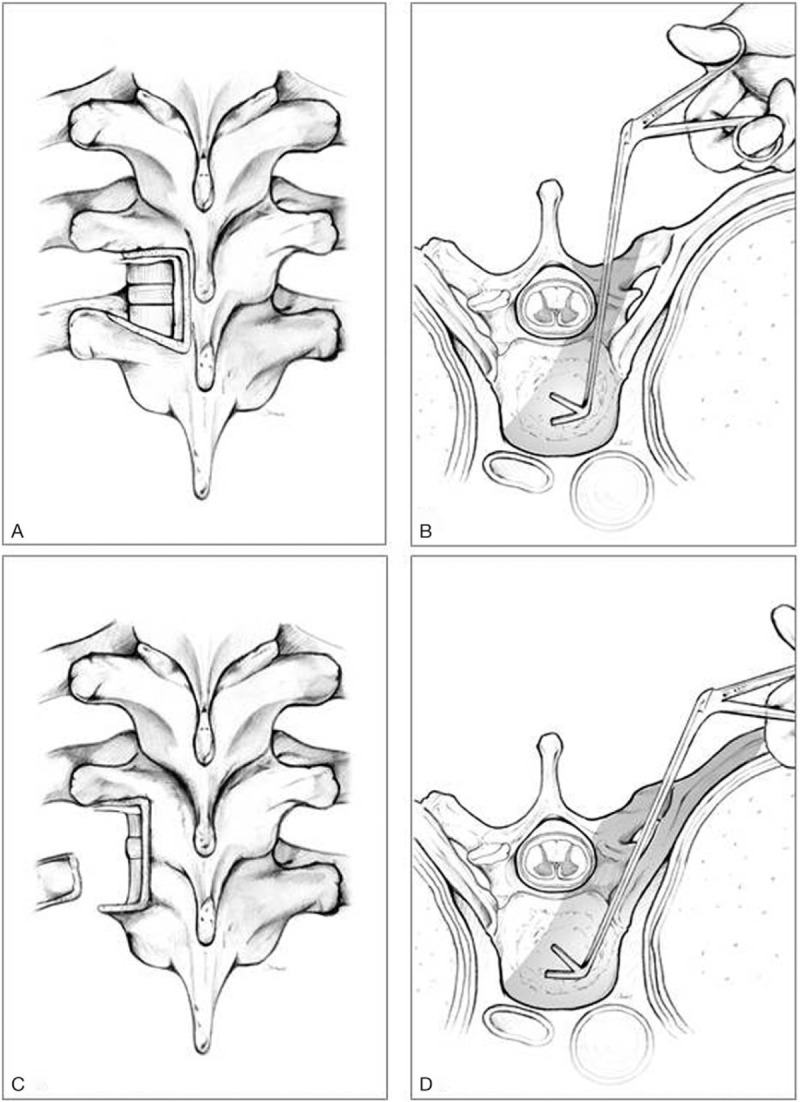
Posterior and axial illustrations, respectively, of the surgical approaches: (A, B) transforaminal thoracic interbody debridement and fusion and (C, D) costotransversectomy. For TTIDF, the pathologic disc lesion is exposed via a transforaminal approach; for costotransversectomy, the rib head and transverse process must be removed in order for the surgical tools to be used far from the dura sac. The gray areas in the 2 axial views (B, D) indicated alternative ways of approaching the disc.

The costotransversectomy procedure was the same as the one described in our previous study,^[7]^ and the TTIDF was a modified method from a previously described transforaminal lumbar interbody debridement and fusion procedure^[8]^ for the thoracic spine. Briefly, once under general anesthesia, the patient was placed on a 4-postspinal frame in the prone position. Pathological lesions in the thoracic spine were identified using portable (C-arm) radiography before starting the procedure. One midline longitudinal skin incision was made over the spinous process, after which the paraspinal muscles were dissected from the spinous process and the lamina. Posterior instrumentation was performed by inserting transpedicle screws into the vertebral bodies 1 or 2 levels above and below the lesion according to the stability and quality of the bone. The side of the vertebral body to which the approach was applied was determined preoperatively according to the severity of bony destruction, the presence of epidural abscesses, and radiculopathy. Allograft cancellous bone with autologous bone chips were impacted into destructive infectious disc space after radical debridement. The schematic diagram of approaching the pathologic disc lesion using 2 different methods is shown in Fig. 1.

Parenteral antibiotics were continued immediately after the operation based on the culture results and suggestions from the infectious disease specialists. Pyogenic spondylodiscitis was first treated with approximately 4 weeks of parenteral antibiotics according to the C-reactive protein (CRP) level, and then followed by oral antibiotics until at least 3 months of total antibiotic therapy (parenteral and oral antibiotics). All patients were encouraged to wear a brace while ambulating for at least 3 months, and they were followed-up postoperatively at 1, 3, and 6 months, and then annually.

For each patient, demographic data, clinical data such as surgical time, the affected level, the instrumented level, intraoperative blood loss and hospital stay, preoperative and postoperative laboratory data including CRP level, white blood cell (WBC) count, and culture reports were collected. In addition, preoperative and postoperative visual analogue scale (VAS) and Oswestry Disability Index (ODI) were also examined. Preoperative magnetic resonance imaging (MRI) scans of the thoracolumbar spine were assessed for diagnosis, and plain radiographs of the thoracolumbar spine before surgery, after surgery, and at follow-up were assessed for kyphosis correction. The kyphosis angle of each lesion site was measured using the superior endplate of the infected vertebral body above and inferior endplate of the infected vertebra below as reference points. Computed tomography (CT) scans were obtained 6 months after surgery to examine the fusion status. Solid fusion was defined as the presence of continuous bridging bone between the vertebral bodies, shown in the sagittal plane; partial union was defined as the presence of cystic lucencies or linear defects between the vertebral bodies within cortical bony bridging. Any bony defect between the vertebral bodies was defined as pseudoarthrosis.

Quantitative variables were expressed as mean ± standard deviation. The study population was divided into 2 groups based on different surgical approaches: 1-stage posterior only approaches (P only group) and anterior debridement and reconstruction with posterior instrumentation during a single anesthesia session (A+P group). The differences between groups were assessed using the *t* test. The threshold of statistical significance was set at *P* < .05. All statistical calculations were performed using SPSS 12.0 software (SPSS Inc., Chicago, IL).

## 3 Results

From November 2009 to October 2014, out of 53 patients who received posterior instrumentation for pyogenic thoracic spondylodiscitis in our orthopedic department, 26 patients were excluded. Among these 26 patients, 7 patients underwent simple posterior decompression, drainage, and long instrumentation for multilevel infectious spondylodiscitis and epidural abscess, 8 patients without severe spinal instability received posterior instrumentation combined with antibiotics treatment, and 11 patients were surgically treated due to postoperative infectious spondylodiscitis. Among the rest of 27 patients who underwent posterior instrumentation with debridement and reconstruction, 2 patients were excluded due to lost to follow-up. The surgeries of 25 enrolled patients were performed by 2 spine surgeons with an average follow-up of 27.3 months. Eighteen patients underwent 1-stage posterior approach (P only group), 12 men and 6 women, and 7 patients received anterior debridement and reconstruction with posterior instrumentation during a single anesthesia session (A+P group), 5 men and 2 women. Among the P only group, 10 patients underwent a costotransversectomy approach with thoracic interbody debridement and fusion, and other 8 patients received TTIDF. One patient in the P only group expired due to colon cancer 14 months after the spinal operation while in hospice care. The demographic, clinical, and laboratory data are summarized in Table 1. Significant differences between the groups were found in the surgical time and hospital stay.

**Table 1:**
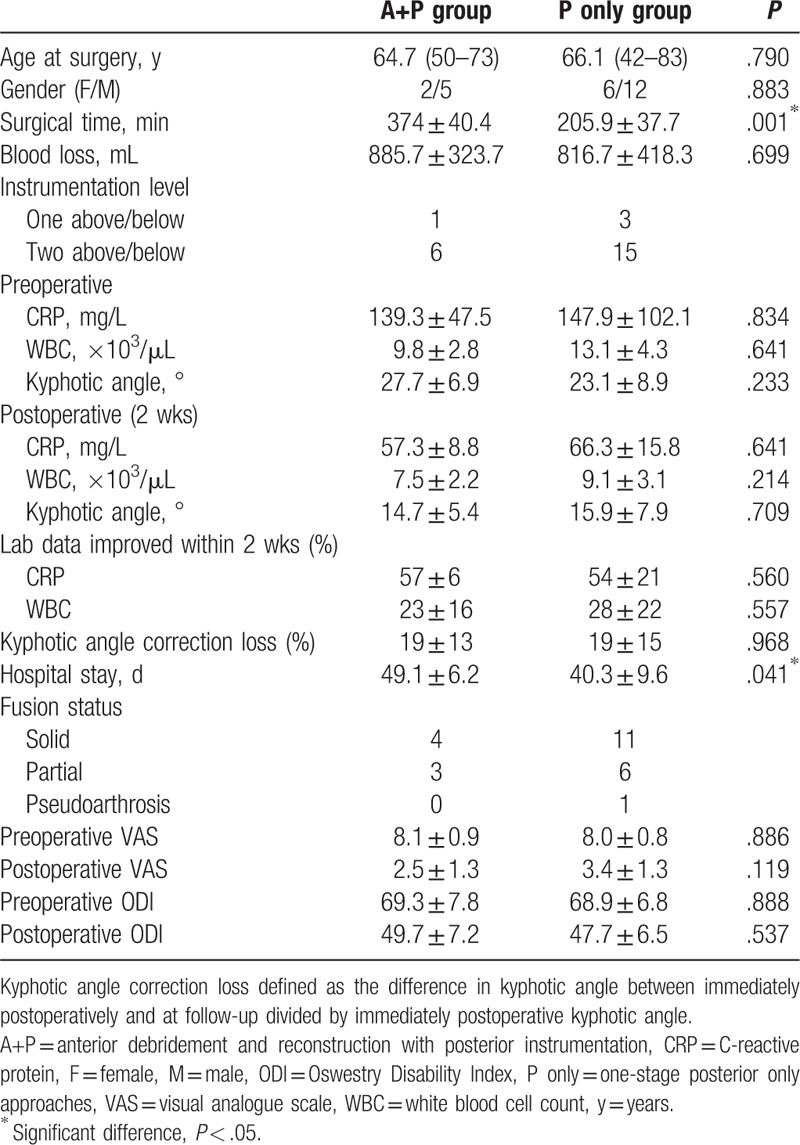
Patient demographic and clinical data.

### 3.1 Infection control

Most patients in the P only group exhibited good infection control and postoperative back pain relief. One patient who underwent TTIDF had recurrent infection with elevated CRP during follow-up and complained of back soreness. The patient was readmitted and the infection was resolved with an additional 4 weeks of antibiotics treatment after ruling out of the possibility of bony destruction and implant loosening. For the P only group, the mean CRP level and WBC count (preoperatively, 147.9 ± 102.1 mg/L and 13.1 ± 4.3 × 10^3^/μL, respectively) were improved 2 weeks postoperatively (66.3 ± 15.8 mg/L and 9.1 ± 3.1 × 10^3^/μL, respectively) (Table 1). The intraoperative culture rate was 83.3% (15/18), with only 3 patients resulted in negative culture. The most common pyogenic pathogen was *Staphylococcus aureus*: 5 methicillin-resistant *S. aureus* and 4 methicillin-sensitive *S. aureus* (Table 2). Four patients had prolonged hospital stay (i.e., more than 6 weeks) due to their slow improvement on the CRP level and WBC count. Concomitant urinary tract infections or pneumonia occurred in 3 patients, while 2 patients had poor surgical wound healing. The infection had subsided in all 18 patients by the time of discharge.

**Table 2:**
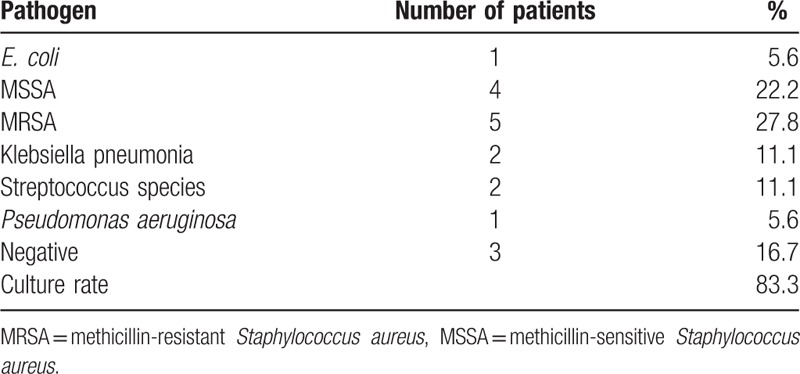
Spine infection culture results of 1-stage posterior approach group.

As the golden standard for the surgical treatment of infectious spondylodiscitis, the A+P group also had good infection control with excellent improvement on the CRP level and WBC count (57 ± 6% and 23 ± 16%, respectively) within 2 weeks after surgery. The intraoperative culture rate was 85.7% (6/7), and *S. aureus* was the most common pathogen. None had recurrent infection, but 2 patients developed postoperative lung atelectasis and fever during hospitalization.

### 3.2 Neurologic status

Nineteen patients had preoperatively complained of severe numbness below the level of the lesion, and all reported improvement after surgery. According to the American Spinal Injury Association (ASIA) Classification, before surgery, the P only group had 8 patients classified as ASIA D and 10 patients were ASIA E, while the A+P group had 4 patients were ASIA D, and 3 patients were ASIA E. All patients were able to walk with crutches or better at discharge, and were classified as ASIA E within 3 months postoperatively. No intraoperative nerve injuries occurred in either group.

### 3.3 Imaging studies

**Figure 2:**
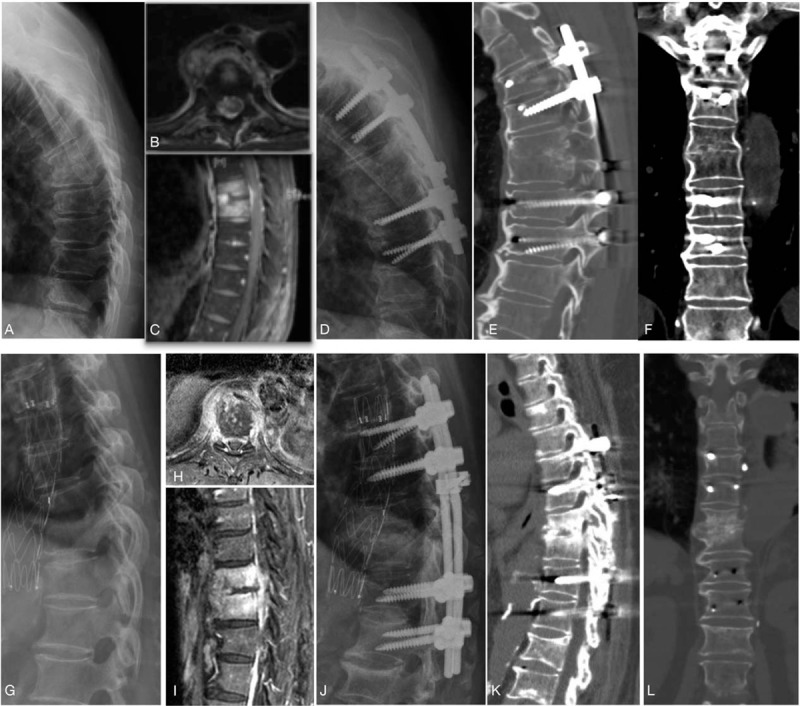
(A–F) Radiographic images of a patient treated T6–7 pyogenic spondylodiskitis with costotransversectomy. (A) Preoperative x-ray showed endplate destruction and local kyphosis at T6-7. (B, C) MRI scans revealed paraspinal abscess acumination and vertebral body signal change. (D) Costotransversectomy with posterior instrumentation T4-9 was performed. (E, F) Postoperative 6-month CT scans revealed solid fusion with continuous cortex and trabecularization across the affected segments. (G–L) Radiographic images of a patient underwent TTIDF for T9-10 pyogenic spondylodiskitis. (G) Preoperative x-ray showed severe endplate destruction and instability at T9-10. (H, I) MRI scans with contrast revealed disc destruction, vertebral body, and para-vertebral enhancement. (J) TTIDF with posterior instrumentation T7-T12 was done. (K, L) Postoperative 6-month CT scans revealed obvious bony bridge and solid fusion.

The fusion status was evaluated by CT scans taken 6 months after surgery (Fig. 2). The overall union rate was 96%, which 17 patients in the P only group and 7 patients in A+P group achieved stable union with either solid union (15 patients) or partial union (9 patients). In the P only group, the mean kyphotic angle was 23.1 ± 8.9° preoperatively, and 15.9 ± 7.9° postoperatively with the mean kyphotic angle correction loss of 19 ± 15%; in the A+P group, the mean kyphotic angle was 27.7 ± 6.9° preoperatively, and 14.7 ± 5.4° with the mean kyphotic angle correction loss of 19 ± 13% (Table 1).

### 3.4 Functional outcomes

All patients reported immediate back pain relief on postoperative day 1. However, when patients in the P only group showed ability to tolerate sitting up on the edge of the bed and attempt ambulation on the same day, patients in the A+P group were not able to attempt ambulation until the removal of chest tube on postoperative day 3 or 4. Both groups achieved improvement in VAS and ODI scores after surgical treatment. The mean VAS score of P only group was improved from 8.0 ± 0.8 to 3.4 ± 1.3 and that of A+P group was improved from 8.1 ± 0.9 to 2.5 ± 1.3. The mean preoperative ODI in P only and A+P group were 68.9 ± 6.8 and 69.3 ± 7.8, respectively, and reduced to 47.7 ± 6.5 and 49.7 ± 7.2 respectively at 3 months postoperatively.

## 4 Discussion

The diagnosis of spinal infection is supported on clinical, physical, laboratory, and imaging findings. In terms of laboratory parameters, CRP level and WBC count are useful parameters that are commonly used to detect infection, and their sensitivity range from 64% to 100% as the markers of postoperative infection.^[9]^ Typically, CRP level shows a downward trend beginning on postoperative day 5.^[10]^ Most patients in this study exhibited dramatic improvement on CRP level and WBC count at 2 weeks postoperatively, which suggested effective infection eradication after surgical debridement.

The mortality rate of infectious spondylodiscitis has been previously reported to be 0% to 11%.^[11,12]^ Most patients with infective spondylodiscitis are also associated with comorbidities such as diabetes mellitus, immunosuppression, intravenous drug use, alcoholism, liver cirrhosis, malignancy, and renal failure.^[13]^ Traditionally, anterior approach with debridement and reconstruction has been regarded as the golden standard for treating infectious spondylodiscitis of the thoracic spine,^[14]^ through either an anterior approach alone or a combination of anterior and posterior approach. Although the anterior approaches allow direct exposure for disc space debridement and ventral column reconstruction, there are many drawbacks, including the possibility of vascular injury, the difficulty of dura repair, relatively high risks of intercostal muscle atrophy, pneumothorax, pneumonia, pleural effusion, chylothorax, and prolonged hospital stay.^[15,16]^ In addition, anterior approaches have been reported to have higher surgery-related morbidity than posterior approaches.^[17]^ In our study, no major postoperative complications or respiratory problems were noted in the P only group. In contrast, greater blood loss, significant longer surgical time, and hospital stay (*P* < .05) with delayed ambulation and rehabilitation were observed in the A+P group; also, 2 patients developed postoperative lung atelectasis and fever, possibly due to respiratory muscle injury, lung parenchyma manipulation and wound pain by rib retraction. Thus, we expected that treating with 1-stage posterior approaches can prevent the major complications associated with the anterior approaches.

Although treating infective thoracic spondylodiscitis via posterior approaches remains controversial, a single-stage posterior approach has many advantages. First, there is no need for changing position during operation, and it requires less operating time and anesthesia time, which is beneficial for both the patient and the surgical team by reducing the risk of complications and the chance of making mistakes. Second, unlike an anterior approach, a posterior approach avoids entry into the pleural space, so without lung manipulation, respiratory muscle damage, or chest tube placement, it has lower risks of vascular injury, postoperative respiratory problems, and morbidity.^[16,18]^ Third, via 2 different posterior approaches mentioned in this study, the interspinous ligament complex and at least 1 side of facet joint are preserved, which help to maintain the stability of the thoracic spine^[19,20]^ and decrease the mechanical failure rate after posterior instrumentation. A single-stage operation also engages patients in early rehabilitation without fear of bone graft loosening or dislodgement between stages.^[5,21]^ Our results showed that patients who treated thoracic spine infection with a single-stage posterior approach achieved similar clinical outcomes of kyphosis angle correction, infection control, functional outcome, and fusion union status when compared with anterior approach, which provided further evidence that a posterior approach with pedicle screw fixation can provide spinal stability to enhance bony union, restore sagittal alignment, prevent the progress of kyphosis, and improve surgical outcomes. The recurrence and posterior spreading of infection are the major concerns with the use of instrumentation in 1-stage posterior approaches; however, several studies have documented the safety of using posterior instrumentation to treat spinal infection, including no increased risk of recurrent infection.^[22,23]^

Compared with the costotransversectomy approach, TTIDF is more similar to a transforaminal approach to the lumbar spine, and has a shorter learning curve. By avoiding the exposure of the retropleural space, TTIDF reduces the risk of iatrogenic pneumothorax and pleural cavity damage; also, it is associated with less soft tissue damage and bony destruction because the rib and transverse process at the infected level are not resected. On the contrary, after radical debridement, autogenous bone chips may not be sufficient for interbody fusion procedure of TTIDF, so the harvest of allogenous bone graft or iliac autogenous bone graft is required.

Both of the 1-stage posterior approaches presented in this study have their drawbacks. The approaches may break up the fascia that acts as a barrier against bacteria and cause severe contamination; however, many spine surgeons have proven that the single-stage posterior radical debridement, reconstruction, and instrumentation with titanium implants was safe and efficacious.^[24–26]^ The ventral spinal column is less clearly to visualize for costotransversectomy and TTIDF than the anterior approaches due to the limited extent. Because of relatively small working space, the posterior approaches are associated with an increased risk of manipulation of the spinal cord and parietal pleural invasion. Moreover, an anterior strut bone graft provides direct support to prevent progressive kyphosis and loss of reduction especially for patients with osteoporosis, but it is unavailable for the single-stage posterior approaches. The size of strut bone graft is too large to impact and significant destruction is needed to access to the entrance. The use of well-impacted cancellous allograft is an alternative to strut bone graft that may avoid donor-site complications and can achieve good clinical outcomes in cervical and lumbar spine arthrodesis.^[27,28]^ In P only group, we carefully preserved the posterior element of the spine and reinforced the stability by pedicle screws fixation with at least 2 levels above and below the lesion for patients with osteoporosis. Therefore, there was no difference observed in loss of correction between the 2 groups at follow-up.

This study has several limitations. First, some degree of sampling bias was present, as it was a retrospective case–control study conducted at a single medical center, especially the number of cases of infection is usually small. The study sample was biased without a larger number of participants, different geographic locations and a wider range of population group. Second, 1-stage posterior-only approaches for treating infectious thoracic spondylodiscitis were technical demanding, and might present some degree of inequivalence among medical centers. Finally, the follow-up period was relatively short. An average of short-term follow-up period for assessing clinical outcomes is usually 2 years.

In conclusion, our results suggested that performing a single-stage posterior approach, either costotransversectomy or TTIDF, for the treatment of thoracic spondylodiscitis can prevent complications and postoperative respiratory problems associated with the anterior approaches. Good clinical outcomes make single-stage posterior approaches preferable to the anterior approaches, especially for patients with medical comorbidities who have less tolerance to long anesthesia time and substantial blood loss. However, a future study with a larger sample size and a longer follow-up period should be conducted to further validate the effectiveness of these single-stage posterior approaches.
